# Retrospective genetic testing (Traceback) in women with early-onset breast cancer after revised national guidelines: a clinical implementation study

**DOI:** 10.1007/s10549-024-07288-9

**Published:** 2024-03-16

**Authors:** Annelie Augustinsson, Niklas Loman, Hans Ehrencrona

**Affiliations:** 1 Care in High Technological Environments, Department of Health Sciences, Lund University, 221 00 Lund, Sweden; 2 Clinical Genetics, Pathology and Molecular Diagnostics, Office for Medical Services, Region Skåne, Lund, Sweden; 3 Oncology, Department of Clinical Sciences in Lund, Lund University, Lund, Sweden; 4 Department of Hematology, Oncology and Radiation Physics, Skåne University Hospital, Region Skåne, Malmö, Sweden; 5 Clinical Genetics, Department of Laboratory Medicine, Lund University, Lund, Sweden

**Keywords:** Breast cancer, Early onset, Genetic testing, Traceback, *BRCA1*, *BRCA2*

## Abstract

**Purpose:**

This study focused on identifying a hereditary predisposition in women previously diagnosed with early-onset breast cancer through a retrospective outreach activity (Traceback). The objectives were to evaluate the possible clinical implementation of a simplified Traceback strategy and to identify carriers of pathogenic variants among previously untested women.

**Methods:**

Three hundred and fifteen Traceback-eligible women diagnosed with breast cancer at 36–40 years in Southern Sweden between 2000 and 2019 were identified and offered an analysis of the genes *ATM*, *BARD1*, *BRCA1*, *BRCA2*, *CHEK2*, *PALB2*, *RAD51C*, and *RAD51D* through a standardized letter. Women who chose to participate were asked about their experiences through a questionnaire. The workload for the study personnel was measured and recorded.

**Results:**

One hundred and seventy-six women underwent genetic testing and pathogenic variants were identified in 9.7%: *ATM* (*n* = 6), *BARD1* (*n* = 1), *BRCA1* (*n* = 3), *CHEK2* (*n* = 5), and *PALB2* (*n* = 2). Women with normal test results were informed through a standardized letter. Carriers of pathogenic variants were contacted by telephone and offered in-person genetic counseling. One hundred and thirty-four women returned the subsequent questionnaire. Most study participants were satisfied with both written pre- and post-test information and many expressed their gratitude. The extra workload as compared to routine clinical genetic counseling was modest (8 min per patient).

**Conclusion:**

The insights from the participants’ perspectives and sentiments throughout the process support the notion that the Traceback procedure is a safe and an appreciated complement to routine genetic counseling. The genetic yield of almost 10% also suggests that the associated extra workload for genetic counselors could be viewed as acceptable in clinical implementation scenarios.

**Supplementary Information:**

The online version contains supplementary material available at 10.1007/s10549-024-07288-9.

## Introduction

Of all breast cancer cases, approximately 5% have been estimated to have strong hereditary backgrounds. The prevalence of pathogenic variants in the specific genes *BRCA1* and *BRCA2* in unselected breast cancer patients has been estimated to be 2–2.5% [1, 2], similarly to what has been reported in the Southern Swedish population [3]. Hereditary predisposition to breast cancer is associated with early onset of the disease [4]. In a recent study based on clinical genetic testing in Sweden over several years, 19% of women with breast cancer at 30–39 years had a pathogenic variant in 1 of 13 analyzed genes [5]. Among women previously diagnosed with breast cancer, the finding of a pathogenic variant is associated with an increased risk for new primary cancers [4, 6, 7]. The identification of pathogenic variants among these women, who would otherwise not have knowledge of their carrier status, is crucial for the prevention of new cancers through increased surveillance and risk-reducing measures. Especially, the inherited risk for ovarian cancer associated with pathogenic variants in *BRCA1* and *BRCA2* is of clinical relevance and has a direct impact on the survival of women treated for *BRCA1*/*2-*associated breast cancers [8]. In addition, knowledge of a pathogenic variant provides opportunities for cancer prevention among healthy family members [9, 10].

For many years, Swedish national breast cancer guidelines have recommended that all women diagnosed with invasive breast cancer at an age of 35 years or younger should be offered a referral for genetic counseling and given the option of genetic testing, regardless of family history of cancer [11]. These recommendations were expanded in 2018 to include all women diagnosed with breast cancer at 40 years or younger [12]. For additional details on current and previous guidelines, see Supplemental 1.

In 2016, the National Cancer Institute held a meeting where a framework for the identification and genetic testing of previously diagnosed but unreferred ovarian cancer patients and other unrecognized carriers of pathogenic variants was discussed and designated ‘Traceback’ [13]. Since then, Traceback among previous ovarian cancer patients and their relatives have been studied [14–17]. However, Traceback among previously diagnosed breast cancer patients has not been thoroughly evaluated. Hence, we conducted a pilot study where a Traceback strategy was evaluated among unreferred women who were diagnosed with breast cancer at the age of 35 years or younger in Southern Sweden between 2000 and 2017 [18]. In this study, 29 women underwent genetic testing and pathogenic variants were identified in four: *BRCA1* (*n* = 2), *CHEK2* (*n* = 1), and *ATM* (*n* = 1).

Because the Swedish national breast cancer guidelines were expanded in 2018 [12], we identified an opportunity to perform a simplified Traceback strategy based on our previous experiences in a larger cohort of women. The primary objective was to evaluate the feasibility of introducing a similar procedure within future clinical routine. We hypothesized that a large percentage of women in this age group would never have received an offer regarding genetic testing and that carriers of breast cancer predisposition pathogenic variants would be identified.

## Materials and methods

### Data collection

**Fig. 1:**
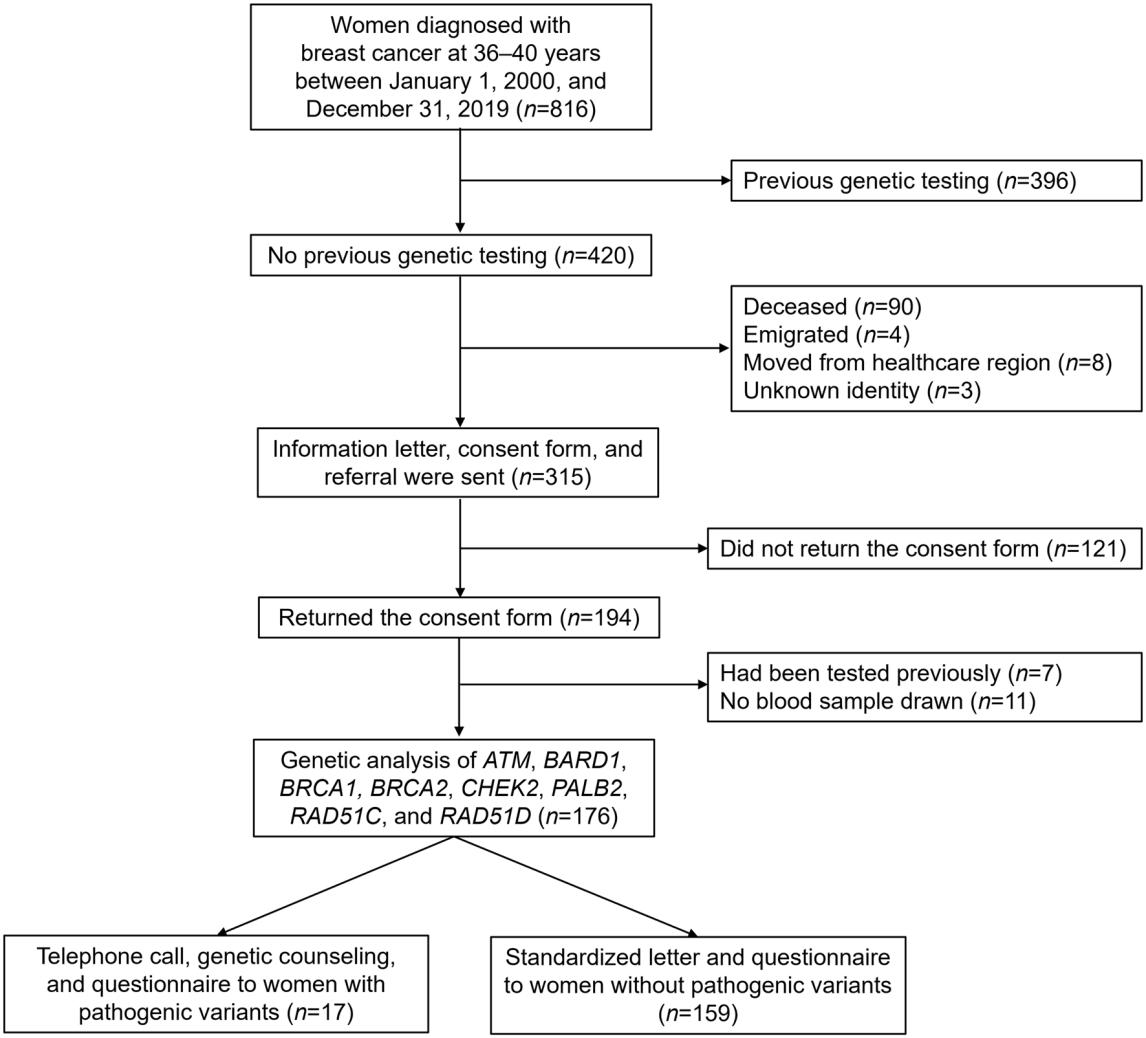
Traceback inclusion: schematic representation of inclusion, exclusion, and genetic testing

National civic registration numbers and information regarding all women who were diagnosed with breast cancer at 36–40 years in the South Swedish Health Care Region between January 1, 2000 and December 31, 2019, (*n* = 816) were retrieved from the Southern Swedish Regional Tumor Registry in Lund and the National Quality Registry for Breast Cancer (NKBC) in Stockholm. Information regarding which patients who had previously undergone genetic testing was retrieved from the Regional Oncogenetic Register (OnkGen) and clinical records at Skåne University Hospital in Lund. Vital status and current addresses were extracted from the Population Register, which is administered by the Swedish Tax Agency. After exclusion of women who had previously undergone genetic testing (*n* = 396) and women who were deceased (*n* = 90), unknown (*n* = 3), emigrated (*n* = 4), or had moved to another healthcare region in Sweden (*n* = 8), an invitation letter was sent to the remaining 315 women (Fig. 1).

### Summary of the Traceback study procedure

The Traceback procedure has previously been described in detail [18]. To summarize, an invitation letter written in Swedish (English translation, Supplemental 2) and a referral form for a blood sample for DNA extraction and analysis of genes linked to inherited increased risk for breast cancer were sent through regular mail. The women consented to study participation and genetic testing by signing a consent form and returning it in an enclosed pre-paid envelope. Subsequently, study participants had a blood sample drawn, without any cost and without a previous meeting with a physician.

When comparing the current Traceback procedure with the Traceback pilot study procedure [18], there were two discrepancies. First, due to updated recommendations in the Swedish national breast cancer guidelines [19], the number of analyzed genes were increased from five to eight (*ATM*, *BARD1*, *BRCA1*, *BRCA2*, *CHEK2*, *PALB2*, *RAD51C*, *RAD51D*). As in the previous study, *TP53* was excluded due to the specific difficulties involved with discussing cancer risks and management with carriers of pathogenic variants in this gene. Second, we simplified the study procedure. We concluded in the pilot study that the follow-up telephone call could be excluded. Hence, only one written reminder was sent after approximately 4 weeks to each of the women who had not returned the consent form.

After the analyses were completed, women with normal test results were notified through a standardized letter (English translation, Supplemental 3). Carriers of pathogenic variants were informed about the test result through a telephone call from a genetic counselor and given time for in-person genetic counseling at the Oncogenetic Clinic in Lund at their earliest convenience. The women had the option to choose between a physical appointment at the outpatient clinic or telephone counseling. Subsequently, a questionnaire with three open-ended questions and six scaled-response questions with Likert rating scales, ranging from 1 (strongly disagree) to 5 (strongly agree), regarding their experiences of the Traceback approach was sent to the women who underwent genetic testing.

To facilitate future discussions regarding the feasibility of clinical implementation of a Traceback procedure, the workload for the study personnel was recorded and subsequently divided per participating woman.

### Statistical analysis

Mann–Whitney *U* test was used to examine the differences in median age at breast cancer diagnosis and study invitation and time between breast cancer diagnosis and study invitation, among women who chose to participate in the study and those who chose not to. Pearson’s Chi-square test was used to examine differences in patient characteristics and between answers to the scaled response questions, as well as between study participation and place of residence. Descriptive statistics are shown as mean ± standard deviation (SD) or median and interquartile range (IQR). All analyses were conducted using the IBM SPSS statistical computing package (version 28.0; SPSS, Inc., Chicago, Illinois, USA). Statistical significance was considered with a two-tailed *P* < 0.05.

## Results

### Previously tested women

A total of 816 women were diagnosed with invasive breast cancer at 36–40 years of age in the South Swedish Health Care Region between January 1, 2000 and December 31, 2019. Out of the 403 women who had previously undergone genetic testing, 79 (19.6%) were carriers of one (or two, *n* = 1) pathogenic variants. The median age at breast cancer diagnosis was 38.7 (IQR 37.3–38.7) years and the median time from diagnosis to genetic analysis was 0.9 years (IQR 0.3–0.9) (Table 1).

**Table 1:** Characteristics of women diagnosed with breast cancer at 36–40 years of age in the South Swedish Health Care Region between January 1, 2000 and December 31, 2019

	All women diagnosed with BC	Women tested previously^a^	Women tested in this study	*P*^b^
Number of patients, *n*	816	403	176	
Age at BC diagnosis, years, median (IQR)	39.1 (37.8–39.1)	38.7 (37.3–38.7)	39.9 (38.5–39.9)	<0.001
Year of BC diagnosis, *n* (%)				<0.001
2000–2009	331 (40.6)	115 (28.5)	83 (47.2)	
2010–2017	381 (46.7)	204 (50.6)	83 (47.2)	
2018–2019	104 (12.7)	84 (20.8)	10 (5.7)	
Time between BC diagnosis and genetic testing, years, median (IQR)	2.2 (0.5–2.2)	0.9 (0.3–0.9)^c^	12.4 (8.9–12.4)	<0.001
Vital status,* n* (%)				
Alive	644 (78.9)	336 (84.2)	176 (100.0)	N/A
Dead	150 (18.4)	60 (15.0)	N/A	
Emigrated	9 (1.1)	5 (1.3)	N/A	
Moved to other healthcare region	10 (1.2)	2 (0.5)	N/A	
Unknown identity	3 (0.4)	N/A	N/A	
PV carriers, *n* (%)				
No PV	477 (83.0)	318 (78.9)	159 (90.3)	N/A^d^
*ATM*	8 (1.4)	2 (0.5)	6 (3.4)	
*ATM* + *CHEK2*	1 (0.2)	1 (0.2)	0 (0.0)	
*BARD1*	1 (0.2)	0 (0.0)	1 (0.6)	
*BRCA1*	34 (5.9)	31 (7.7)	3 (1.7)	
*BRCA2*	26 (4.5)	26 (6.5)	0 (0.0)	
*CHEK2*	16 (2.8)	11 (2.7)	5 (2.8)	
*PALB2*	3 (0.5)	1 (0.2)	2 (1.1)	
*TP53*	4 (0.7)	4 (1.0)	N/A	
Other	3 (0.5)	3 (0.7)	N/A	
Unknown PV	2 (0.3)	2 (0.5)	N/A	
Unknown result	4 (0.7)	0 (0.0)	N/A	
Missing/not tested (*n*)	237			

*Abbreviations*: *BC* breast cancer, *IQR* interquartile range, *PV* pathogenic variant

^a^Including the seven women who were invited to participate in the study but had already been tested

^b^Mann–Whitney *U* test or Pearson’s Chi-square test was used to examine differences between women who were tested previously and women who were tested in this study

^c^Date for genetic testing was missing for 27 women who were tested previously

^d^The carrier numbers are not comparable since not all women who were tested previously were tested for the same genes as in the current study

### Study population

Three hundred and fifteen women were offered an analysis of the genes *ATM*, *BARD1*, *BRCA1*, *BRCA2*, *CHEK2*, *PALB2*, *RAD51C*, and *RAD51D*. The median ages at breast cancer diagnosis and study invitation were 39.8 (IQR 38.5–39.9) and 51.5 (IQR 46.7–57.1) years, respectively. The median time from diagnosis to study invitation was 12.1 (IQR 8.3–17.9) years (Table 1).

### Consent, genetic testing, and variant detection

Of the 315 invited women, 147 (46.7%) women returned the signed consent form within 4 weeks. Subsequently, 162 written reminders were sent, which resulted in an additional 47 (14.9%) consent forms being returned. Of the 194 women who accepted participation, 176 (55.9% of the entire cohort, 90.7% of the women who consented) subsequently had a blood sample drawn for DNA extraction and genetic analysis. The median turn-around time for the laboratory analysis, i.e., the time from blood sample registration to clinical report delivery, was 22 (IQR 21–24) days. Pathogenic variants were identified in 17 (9.7%) women: 6 in *ATM*, 1 in *BARD1*, 3 in *BRCA1*, 5 in *CHEK2*, and 2 in *PALB2* (Supplemental 4). Variants classified as pathogenic (class 5) or likely pathogenic (class 4) are collectively called pathogenic variants in this paper.

Out of the 139 women who did not undergo genetic testing, 7 had already been tested elsewhere, 1 did not want to, 1 was too anxious to, and 1 was too ill to participate. Three women had their information letter returned (address unknown). The remaining 126 (90.6%) women did not communicate a reason for choosing not to participate.

There were no statistically significant differences between median age at breast cancer diagnosis (39.9 vs. 39.4; *p* = 0.26) or study invitation (51.7 vs. 51.2 years; *p* = 0.18) of the women who participated in the Traceback study compared with those who did not, nor was there a significant difference between median time from breast cancer diagnosis to study invitation (12.2 vs. 11.8 years; *p* = 0.31). In addition, when analyzing the association between participation in the Traceback study and place of residence, no significant difference was found (*p* = 0.55).

### Workload assessment

As compared to clinical routine, the additional tasks for the Traceback procedure consisted of registry matching and sending invitation letters, referral forms, and reminders. Between August 9, 2022 and November 10, 2022, between 10 and 40 invitation letters with referral forms were sent on a weekly basis (depending on the workload at the laboratory) and between September 1, 2022 and December 12, 2022, approximately 10–20 reminders were sent each week. This work was recorded, and a total time of 24 h was noted, corresponding to 4.6 min per invited woman or 8.2 min per woman who underwent genetic testing.

### Questionnaire

Of the 176 women who underwent genetic testing, 134 (76.1%) returned the subsequent questionnaire. Study participants’ answers to the scaled-response questions are shown as mean ± SD (Table 2). Most women, both with and without pathogenic variants, reported that they understood and were satisfied with the written study information, as well as with the opportunity for additional contacts and going through with the genetic testing. Five (4.2%) of the women with normal test results and two (14.3%) of the carriers of a pathogenic variant would have wanted additional oral information. Most women reported that they had shared the information with their relatives and that they would recommend a female friend with breast cancer to undergo genetic testing in the same way that they did.

**Table 2:** Closed-ended scaled-response questions with answers ranging from 1 (strongly disagree) to 5 (strongly agree)

	Women with normal test results	Women with a pathogenic variant	*P*^a^
Number of patients, *n*	120	14	
Questions, mean (SD)			
I understood the obtained written study information	4.79 (0.447)	4.86 (0.363)	>0.3
I am satisfied with the obtained written study information and the opportunity for further contacts	4.85 (0.513)	4.86 (0.363)	>0.3
I would have wished for additional oral information	1.64 (0.877)	2.29 (0.994)	0.08
I am satisfied with undergoing genetic testing	4.93 (0.250)	5.00 (0.000)	>0.3
I have shared the obtained information with my relatives	4.20 (1.120)	4.57 (0.756)	>0.3
I would recommend a female friend with breast cancer to undergo genetic testing in the same way that I did^b^	4.72 (0.801)	4.93 (0.267)	>0.3

^a^Pearson’s Chi-square test was used to examine differences between answers to the scaled-response questions

^b^Two women with normal test results did not answer this question

The first open-ended question was ‘*What was your experience of being offered genetic testing through a letter?*’. To this question, 112 (83.6%) women had positive responses. These responses included some short answers, such as ‘*Positive*,’ ‘*Very positive*,’ ‘*Good*,’ ‘*Very good*,’ ‘*Totally OK*,’ and ‘*Very good, grateful for the offer*.’ The majority of participants wrote longer answers, such as ‘*Very positive, I was immensely happy to feel that I am not forgotten*.’ ‘*I was very happy to get the offer, a very simple way to do the testing*.’ ‘*I was glad to be elected to collaborate with the investigation*,’ and ‘*My first thought was that it is good that doctors, researchers, etc. offer and do this type of studies on women*.’ Twenty (14.9%) women reported mixed feelings, e.g., ‘*I was hesitant at first, but after a discussion with my family I realized that it was only beneficial*’ and ‘*Exciting and scary at the same time, you both want to know and are afraid to know*.’ One woman wrote that she was ‘*Surprised. Made me ponder what I would want to know*.’ One woman had a negative response and wrote that it was ‘*A bit hard, almost shocking. Almost as hard as getting the cancer diagnosis*.’

When answering the question ‘*What was the main reason for choosing to participate in the study?*’, 90 (67.2%) women reported only one reason. The remaining 44 women reported two (or more) reasons. In total, 71 (53.0%) women reported that they wanted increased knowledge for themselves. One woman wrote ‘*I have wondered if I have any of the genes that can cause breast cancer, but I have not known how to find out if I am a carrier*.’ Five women wrote that they wanted to be able to receive extended surveillance or additional interventions, such as prophylactic removal of the contralateral breast. Seventy-four (55.2%) women reported that they wanted to know more for the sake of their family members, i.e., the hereditary aspect of the disease. Illustrative quotes are ‘*To know if it was hereditary, for my daughter’s sake*’ and ‘*That I do not have anything that I will pass on to my daughter*.’ Thirty-three (24.6%) women had altruistic reasons and reported that they wanted to support medical research or help others.

Women with normal test results (*n* = 120) subsequently answered the question ‘*What was your experience of being informed of the result from the genetic analysis through a letter?*’. One hundred and twelve (93.3%) women had positive responses, such as ‘*It was good, then you can read it many times*’, ‘*I was very happy to receive the negative result in a letter*’, and ‘*It felt natural since the study information was delivered by mail*.’ Six women reported mixed feelings, e.g., ‘*Totally OK. A bit nervous to open it in the beginning, but when I read the result then it was only joy*.’ One woman wrote that she thought it was hard and one woman answered that she had not received the result. Women with pathogenic variants (*n* = 14) answered the question ‘*What was your experience of being informed of the result from the genetic analysis through a telephone call and subsequent genetic counseling?*.’ Ten (71.4%) women had a positive answer, such as ‘*I think it worked well. The physician was distinct, and it felt like he took the time to answer our questions*.’ and ‘*Good. I got a long and informative talk that gave room for questions. Pertinent information*.’ Two women expressed criticism regarding the counseling process, with differing views: ‘*I think it would be better to get the result by mail and not a telephone call, but maybe you would want to talk with someone after you have read the letter*’ and ‘*Not so good. I wanted to have a physical meeting instead. I think that it will be better, will be less misunderstandings*.’ Two women reported that they felt shocked and sad about the genetic test result.

At the end of the questionnaire, 54 (40.3%) women wrote additional comments, such as being thankful (*n* = 42), being supportive of the study (*n* = 9), or feeling happy or safe (*n* = 3). Furthermore, three women wrote that they would have wanted to get the offer of genetic testing earlier, i.e., not years after their initial breast cancer diagnosis.

## Discussion

Retrospective identification of carriers of pathogenic variants among previously treated cancer patients is a challenging undertaking from a practical, ethical, and psychological perspective. At the same time, it has the potential to reduce serious morbidity and probably even save lives among women that were not identified as carriers of pathogenic variants in association with a previous cancer diagnosis. We conducted a Traceback study to retrospectively identify carriers of pathogenic variants among women diagnosed with early-onset breast cancer to assess the feasibility and effectiveness of this approach. The study aimed to contribute insight into the potential benefits of expanding the Traceback procedure clinically, with results on the prevalence of pathogenic variants in this setting, the effectiveness of outreach strategies, and the experiences and motivations regarding retrospective genetic testing.

The current Traceback study employed a proactive outreach strategy to women who were diagnosed with breast cancer between 36 and 40 years in the South Swedish Health Care Region who had not previously undergone genetic testing. The study participation rate was encouraging, with 46.7% of invited women returning signed consent forms within 4 weeks, and an additional 14.9% returning them after receiving reminders. The outreach approach successfully engaged most of the target population, leading to 55.9% of those invited undergoing genetic testing. This suggests that offering genetic testing to previously unreferred breast cancer patients through a mail-based approach is a viable and effective method. Furthermore, the workload on the genetic counseling staff was manageable, with an estimated eight additional minutes of work for every woman who accepted to participate, as compared to clinical routine. However, the workload per patient will depend on the structure of the healthcare system, and the easy access to public registries in Sweden has facilitated the current study. Of the women who chose not to participate in the study, the majority (90.6%) did not communicate their reason, indicating a need for further research to understand barriers to genetic testing.

It is well established that knowledge of carriership is of clinical importance, since increased surveillance and prophylactic surgery leads to significantly reduced morbidity and mortality [4, 6, 20], especially for women harboring pathogenic variants in *BRCA1*, *BRCA2*, or *PALB2*. Out of the early-onset breast cancer patients who had previously undergone genetic testing, 19.6% were carriers of pathogenic variants in *ATM*, *BARD1*, *BRCA1*, *BRCA2*, *CHEK2*, or *PALB2*. In the current study, 9.7% of the women were identified as carriers of pathogenic variants in these six breast cancer susceptibility genes. This highlights the significant number of women who may carry pathogenic variants but have not been identified due to previous more restrictive testing criteria. These results stress the importance of expanding genetic testing efforts among early-onset breast cancer patients, as identification of pathogenic variants in this population is important not only for individual risk assessment but also for guiding preventive strategies and informing family members.

Out of the women who had not previously undergone genetic testing, 21.4% were deceased. Potentially, several of these women were unknown carriers of a pathogenic variant. In such circumstances, the carrier status is likely to remain unknown until a subsequent cancer diagnosis in a family member might trigger testing.

In a recent Swedish publication, the percentage of carriers of a pathogenic variant in *BRCA1* or *BRCA2* among women diagnosed with breast cancer at 40 years or younger was 10.0% (105/1055 patients) [5]. For the women identified in the current study, who were previously tested and therefore excluded from the Traceback procedure, the corresponding percentage was 14.2% (57/375 women) (Table 1). Three of the women who were tested in the current study were carriers of pathogenic variants in *BRCA1* and none in *BRCA2*, a figure that might seem low in comparison. While this discrepancy may be due to chance, it is also possible that women with a strong family history or other risk factors compatible with a high increased breast (and ovarian) cancer risk were more likely to be offered testing early [11], explaining the lower yield in the Traceback cohort.

The questionnaire responses provided insights to the experiences and motivations of women who underwent genetic testing. Most participants reported an understanding of, and satisfaction with, the study information, emphasizing the importance of clear and concise communication in the Traceback procedure. Further support for the simplified procedure was given by a previous study, BRCAsearch, where 818 newly diagnosed breast cancer patients were contacted in a similar manner, resulting in very few telephone calls to the designated genetic counselor and a general high level of satisfaction with the procedure [3, 21]. It is notable that most participants wanted to gain knowledge not only for themselves but also for the sake of their family members, highlighting the hereditary aspect of the disease, which is concordant with findings in previously published studies [18, 22]. Importantly, many women expressed a willingness to share information with their relatives and would recommend genetic testing to others. The differences in experiences between women who received their results through a standardized letter and those who received a telephone call from a genetic counselor and subsequent genetic counseling suggest that personalized communication may be preferred when delivering potentially life-altering genetic information. Providing support and counseling for carriers of pathogenic variants is essential to help individuals navigate their as well as family members’ risks and make informed decisions regarding surveillance and prevention.

The backdrop of the current study was that the Swedish breast cancer guidelines were amended to include a recommendation of genetic testing for all women with breast cancer at age 40 years or younger. It is important to recognize that guidelines evolve over time, and in the recently published ASCO—Society of Surgical Oncology Guidelines [23], the authors recommend that genetic testing of *BRCA1* and *BRCA2* should be offered to all patients with a previous history of breast cancer and without active disease diagnosed at 65 years or younger, as well as selectively to patients diagnosed after 65 years if the result will inform personal risk management and/or family risk assessment. In addition, testing for high-penetrance genes beyond *BRCA1* and *BRCA2* should be offered to those with a family history, and, if necessary, for moderate-penetrance genes when coupled to personal and family cancer risk information. Such changes in guidelines and recommendations highlight the need for the development of cost-effective clinical retrospective testing strategies.

This study has some limitations. The sample size was limited. The reasons for non-participation among eligible women were not elucidated, which would be advantageous to explore in future research to improve outreach strategies. However, the Swedish Ethical Review Authority does not approve contact with individuals who decline research consent, making such studies difficult.

The findings of this Traceback study have several implications for clinical practice. The procedure was well accepted by participants, led to an additional genetic yield of almost 10% in this previously untested group of women, and should be seen as cost-efficient considering the modest additional workload compared to the current clinical routine. An alternative to the Traceback approach would be to identify patients as a part of clinical follow-up at the respective departments where this is being taken care of. However, given the considerable time that had elapsed since the primary diagnoses in this study and the fact that most healthy individuals would probably no longer have an active contact with the healthcare unit where primary care was provided, the possibility to reach out to these individuals in the clinical context would probably be limited. When considering implementation of similar protocols within healthcare, there is a need for continued efforts to educate both medical staff and patients about the benefits of genetic testing for breast cancer risk. Further research is needed to explore barriers to genetic testing and refine interventions that address the diverse needs of this population.

### Supplementary Information

Below is the link to the electronic supplementary material.

Supplementary file1 (DOCX 19 KB)

Supplementary file2 (DOCX 31 KB)

Supplementary file3 (DOCX 28 KB)

Supplementary file4 (DOCX 29 KB)

## Data Availability

The datasets generated during and/or analyzed during the current study are not publicly available due to privacy/ethical restrictions but are available from the corresponding author on reasonable request.
