# Supplementary material for: Retrospective genetic testing (Traceback) in women with early-onset breast cancer after revised national guidelines: a clinical implementation study
Source: Breast Cancer Res Treat. 2024 Mar 16;205(3):599–607. doi: 10.1007/s10549-024-07288-9 (PMC11101361; doi:10.1007/s10549-024-07288-9)
Supplement: Supplementary file 1 — Supplementary file1 (DOCX 19 KB) [file 10549_2024_7288_MOESM1_ESM.docx]

Supplemental 1: Summarized Swedish national breast cancer recommendations for oncogenetic testing

Genetic testing should be recommended if the patient fulfills at least one of the following criteria.

a) Current (2023) guidelines^1^

| Any of the following |
| --- |
| Breast cancer ≤40 years of age. |
| Breast cancer ≤50 years of age, if there is at least one additional case of breast cancer in first- or second-degree relatives in the same family branch. Bilateral breast cancer counts as two cases. The second case can also be ovarian or tubal cancer, early-onset prostate cancer (≤65 years of age), or pancreatic cancer. |
| Breast cancer ≤60 years of age, if there are at least two additional cases of breast cancer in first- or second-degree relatives in the same family branch. Bilatral breast cancer counts as two cases. The other cases can also be ovarian or tubal cancer, early-onset prostate cancer (≤65 years of age), or pancreatic cancer. |
| Triple-negative breast cancer regardless of age. |
| Male breast cancer regardless of age. |
| Ovarian cancer including tubal cancer and primary peritoneal carcinomatosis (non-borderline) regardless of age. |

b) Previous (2014) guidelines^2^

| Any of the following^*^ |
| --- |
| Breast cancer ≤35 years of age. |
| Breast cancer ≤40 years of age, if there is at least one additional case of breast cancer in first-degree relatives or second-degree relatives through a male. The second case can also be ovarian cancer. |
| Breast cancer ≤50 years of age, if there are at least two additional cases of breast cancer in first-degree relatives or second-degree relatives through a male. The other cases can also be ovarian cancer. |
| Breast cancer and ovarian cancer in the same individual regardless of age. |
| Male breast cancer regardless of age. |

^*^Bilateral breast cancer, triple-negative breast cancer, prostate cancer, and pancreatic cancer strengthens the indication to

consider testing.

**References**

1. Regionala cancercentrum i samverkan (2023) Nationellt vårdprogram bröstcancer, version 4.3.

2. Regionala cancercentrum i samverkan (2014) Nationellt vårdprogram bröstcancer, version 1.0.

Retrospective genetic testing (Traceback) in women with early-onset breast cancer after revised national guidelines – a clinical implementation study

Breast Cancer Research and Treatment

Annelie Augustinsson^1,2,3^*, Niklas Loman^3,4^, and Hans Ehrencrona^2,5^

^1^Care in High Technological Environments, Department of Health Sciences, Lund University, Lund, Sweden ^2^Clinical Genetics, Pathology and Molecular Diagnostics, Office for Medical Services, Region Skåne, Lund, Sweden
^3^Oncology, Department of Clinical Sciences in Lund, Lund University, Lund, Sweden
^4^Hematology, Oncology and Radiation Physics, Region Skåne, Malmö, Sweden
^5^Clinical Genetics, Department of Laboratory Medicine, Lund University, Lund, Sweden

*Corresponding author: annelie.augustinsson@med.lu.se
