# Supplementary material for: Retrospective genetic testing (Traceback) in women with early-onset breast cancer after revised national guidelines: a clinical implementation study
Source: Breast Cancer Res Treat. 2024 Mar 16;205(3):599–607. doi: 10.1007/s10549-024-07288-9 (PMC11101361; doi:10.1007/s10549-024-07288-9)
Supplement: Supplementary file 2 — Supplementary file2 (DOCX 31 KB) [file 10549_2024_7288_MOESM2_ESM.docx]

**Supplemental 2: Invitation letter**

Why do not all young women with breast cancer receive genetic counseling?

**A research study with an offer of genetic testing for women who were diagnosed with breast cancer at an early age.**

**Offer of genetic testing**

Breast cancer early in life increases the probability of finding a hereditary cause for the diagnosis. Within this research project, we study all women in the South Swedish Health Care Region who have been diagnosed with breast cancer at an age of 40 years or younger and who previously have not been in contact with the Oncogenetic Clinic in Lund. Given that you are one of the above-described women, you are now offered the possibility of having genes which are linked to an inherited increased risk for breast cancer analyzed through a common blood test.

**Background and objective**

Annually, approximately 8,000 women are diagnosed with breast cancer in Sweden. Out of these, approximately 400 (5%) women are younger than 40 years when they receive their diagnosis. Breast cancer is in most cases not coupled to a strong heredity. In some families, you may however find alterations in genes (mutations) which can explain why an individual develops breast cancer. If you have such a mutation, it can affect how the breast cancer should be treated and followed up.

Most often, individuals with mutations have inherited it from one of their parents. If so, there are also often breast cancer cases among relatives, but this is not always the case. Relatives of a person with a mutation can be given the option of genetic testing and, if necessary, take preventive measures if it is shown that they too have the inherited increased risk.

To be diagnosed with breast cancer early in life increases the probability of finding a hereditary cause for the diagnosis. Hence, it is recommended in the Swedish national breast cancer guidelines since 2018 that all women who have been diagnosed at an age of 40 years or younger should be offered a referral to an oncogenetic clinic for genetic counseling and subsequently have the option of analysis of genes linked to suspected hereditary breast cancer. If you were diagnosed with breast cancer before 2018, previous recommendations were different, which can be an explanation if you have not previously been offered genetic analysis.

**Research project**

In this research project, where data has been collected from the Regional Cancer Center, the National Quality Register for Breast Cancer, and the Oncogenetic Clinic in Lund, we are studying all women in the South Swedish Health Care Region (i.e., Skåne, Blekinge, Kronoberg, and Southern Halland) who have been diagnosed with breast cancer at an age of 40 years or younger between the years 2000 and 2019 and previously have not been in contact with the Oncogenetic Clinic in Lund.

Given that you are one of these women, you are now offered the possibility to analyze genes linked to an inherited increased risk for breast cancer through a common blood sample (one EDTA-vial with 7 mL of blood). In the genetic analysis, the genes *ATM*, *BARD1*, *BRCA1,* *BRCA2*, *CHEK2*, *PALB2*, *RAD51C*, and *RAD51D* are included, since previous research has shown that approximately one in ten women (more than 10%) in your situation carry an inherited mutation in any of these genes. If a mutation is found, it is most commonly seen in one of the genes *BRCA1* or *BRCA2.*

If a mutation is found in you, you will be referred to the Oncogenetic Clinic in Lund for subsequent information about how it could possibly affect your own and your relatives’ care.

**What are the consequences if one has a mutation?**

Healthy women with a mutation in *BRCA1* or *BRCA2* have a high risk of being diagnosed with breast cancer. Among women without a mutation, the risk is approximately one in ten to be diagnosed with breast cancer throughout their lifetime, i.e., approximately 10%. If one has a mutation in *BRCA1* or *BRCA2*, the risk is instead approximately five to eight in ten, i.e., 50–80%. A mutation in certain other genes can lead to a more moderate increased risk.

Women with a mutation who have already had breast cancer do not have an increased risk of recurrence of the disease compared with women without a mutation. However, women with a mutation have a higher risk of being diagnosed with a new breast cancer in the same breast or in the other breast. Hence, these women are followed up extra carefully with breast examinations. One can also opt for prophylactic surgery of the healthy breast, where reconstruction can be done in the same way as among healthy women.

Female carriers of mutations in *BRCA1* and *BRCA2* also have an increased risk for ovarian cancer, where up to half of the women are affected throughout life. This risk is much higher than in the general population, where the risk for ovarian cancer is approximately 1–2% (one to two women in one hundred). To prevent ovarian cancer, *BRCA* mutation carriers are recommended prophylactic surgery of the ovaries after childbearing is finished. *PALB2*, *RAD51C*, and *RAD51D* mutation carriers have a moderate increased risk for ovarian cancer, and especially if ovarian cancer is present in the family, women with such a mutation could also opt for prophylactic surgery.

If a mutation is found in any of the genes *ATM*, *BARD2* or *CHEK2,* it is not associated with a clear increased risk of cancer in any other organ than the breasts.

**What does this mean to you?**
If you consent to the genetic analysis, you sign the informed consent for participating in the study (the pink paper) and return it to us in the enclosed envelope. Subsequently, you bring the blood test referral form which is enclosed in the letter to the hospital or local health center, where the blood sample will be drawn without a previous meeting with a physician. The analysis then takes up to 3 months to complete. If you wish to have further information before you decide on whether you want to participate in the study or not, contact the principal investigator Annelie Augustinsson (see contact information below).

If we have not received an answer from you within 3 weeks after sending this information letter, we will contact you again through a follow-up letter. Women who leave a blood sample could also be contacted with questionnaires or interview as a part of the study.

It does not cost anything to participate in this study, but neither is any economic compensation given for the participation.

**If a mutation is not found during analysis**If you are not a carrier of a mutation, we will notify you with a letter through regular mail.

In most cases this means that the likelihood for a strong heredity for breast cancer is very low and that subsequent investigation is not motivated. Any daughters, sisters, and other female relatives should then, just as all other woman, be encouraged to attend regular mammographic examinations from 40 years of age, within the so-called screening program.

In very rare cases, there could exist a strong heredity caused by other genes than the ones that have been analyzed within the study. You are always welcome to contact the Oncogenetic Clinic in Lund if you have any remaining questions or additional information about your relatives that you would like to discuss.

**If a mutation is found during analysis**

If we find that you are a carrier of a mutation, we will call you on the telephone number that you have specified in the informed consent form, and you will then be offered a visit to the Oncogenetic Clinic in Lund for further information. On this visit, you are welcome to bring a next of kin or relative. If a physical visit in Lund does not suit you, we can also arrange a meeting via telephone or video call.

**Are there any risks associated with participating in the study?**

There are no medical risks. However, the knowledge of being a mutation carrier could cause feelings of anxiety or depression, which are usually transitory. For instance, one could feel sad about the risk that one’s children might have inherited the mutation.

**Are there any advantages associated with participating in the study?**

If it turns out that you belong to the group of women who have been diagnosed with breast cancer and have a mutation, it is an advantage to be aware of it. This means that you could take prophylactic measures to decrease the risk for additional breast cancer or ovarian cancer through extra surveillance and eventual prophylactic surgery.

**Biobank, data, and confidentiality**

Your blood sample and the results from the genetic analysis will be stored at a biobank (BD41) at Clinical Genetics and Pathology, Region Skåne, until further notice. Your answers and results will be handled so that unauthorized personnel cannot access them. The sample could be used in future research, but only after a new ethical approval.

Region Skåne is responsible for your personal data. These will be handled according to the EU’s General Data Protection Regulation (GDPR). You have the right to obtain information about the personal data that has been registered about you annually, and if required have possible errors corrected. You can also request that your blood sample must not be used in the future, that information about you should be erased or that the handling of your personal data should be restricted.

If you want to take part of the information, you can contact principal researcher Annelie Augustinsson (see contact information below). If you are dissatisfied with how your personal data is handled, you have the right to send a complaint to the Data Protection Officer, which is a supervisory authority. The Data Protection Officer can be reached at the address Region Skåne, SE-291 89 Kristianstad.

Participation in this study is voluntary and you can discontinue your participation at any chosen time. If you choose not to participate or want to discontinue your participation, you do not need to specify any reason and it will not affect your future care or treatment.

For further information or if you have questions about the study, please contact:

Annelie Augustinsson
Faculty of Medicine
Lund University

The entity principally responsible for research and personal data is Region Skåne.
Principal researcher is Annelie Augustinsson, PhD in Medical Science.
Responsible researcher at the Oncogenetic Clinic is Dr. Hans Ehrencrona, Senior Consultant and Associate Professor.

Retrospective genetic testing (Traceback) in women with early-onset breast cancer after revised national guidelines – a clinical implementation study

Breast Cancer Research and Treatment

Annelie Augustinsson^1,2,3^*, Niklas Loman^3,4^, and Hans Ehrencrona^2,5^

^1^Care in High Technological Environments, Department of Health Sciences, Lund University, Lund, Sweden ^2^Clinical Genetics, Pathology and Molecular Diagnostics, Office for Medical Services, Region Skåne, Lund, Sweden
^3^Oncology, Department of Clinical Sciences in Lund, Lund University, Lund, Sweden
^4^Hematology, Oncology and Radiation Physics, Region Skåne, Malmö, Sweden
^5^Clinical Genetics, Department of Laboratory Medicine, Lund University, Lund, Sweden

*Corresponding author: annelie.augustinsson@med.lu.se
