# Supplementary material for: Retrospective genetic testing (Traceback) in women with early-onset breast cancer after revised national guidelines: a clinical implementation study
Source: Breast Cancer Res Treat. 2024 Mar 16;205(3):599–607. doi: 10.1007/s10549-024-07288-9 (PMC11101361; doi:10.1007/s10549-024-07288-9)
Supplement: Supplementary file 4 — Supplementary file4 (DOCX 29 KB) [file 10549_2024_7288_MOESM4_ESM.docx]

**Supplemental 4: Clinically relevant variants detected in the Traceback study**

| **Gene** | **Transcript** | **HGVSc** | **HGVSp** | **Classification** | ***n*** |
| --- | --- | --- | --- | --- | --- |
| *ATM* | NM_000051.4 | c.3245_3247delinsTGAT | p.(His1082Leufs*14) | Pathogenic (5) | 1 |
| *ATM* | NM_000051.4 | c.7764dup | p.(Lys2589*) | Likely pathogenic (4) | 1 |
| *ATM* | NM_000051.4 | c.7872T>A | p.(Cys2624*) | Likely pathogenic (4) | 1 |
| *ATM* | NM_000051.4 | c.331+5G>A | p.? | Likely pathogenic (4) | 1 |
| *ATM* | NM_000051.4 | c.497-2A>G | p.? | Likely pathogenic (4) | 1 |
| *ATM* | NM_000051.4 | c.3993+1G>A | p.? | Pathogenic (5) | 1 |
| *BARD1* | NM_000465.4 | c.1476T>A | p.(Tyr492*) | Likely pathogenic (4) | 1 |
| *BRCA1* | NM_007294.4 | c.3048_3052dup | p.(Asn1018Metfs*8) | Pathogenic (5) | 1 |
| *BRCA1* | NM_007294.4 | c.1687C>T | p.(Gln563*) | Pathogenic (5) | 1 |
| *BRCA1* | NM_007294.4 | c.2296_2297del | p.(Ser766*) | Pathogenic (5) | 1 |
| *CHEK2* | NM_007194.4 | c.1100del | p.(Thr367Metfs*15) | Pathogenic (5) | 4 |
| *CHEK2* | NM_007194.4 | c.444+1G>A | p.? | Pathogenic (5) | 1 |
| *PALB2* | NM_024675.4 | c.2167_2168del | p.(Met723Valfs*21) | Pathogenic (5) | 1 |
| *PALB2* | NM_024675.4 | c.2074C>T | p.(Gln692*) | Pathogenic (5) | 1 |

Retrospective genetic testing (Traceback) in women with early-onset breast cancer after revised national guidelines – a clinical implementation study

Breast Cancer Research and Treatment

Annelie Augustinsson^1,2,3^*, Niklas Loman^3,4^, and Hans Ehrencrona^2,5^

^1^Care in High Technological Environments, Department of Health Sciences, Lund University, Lund, Sweden ^2^Clinical Genetics, Pathology and Molecular Diagnostics, Office for Medical Services, Region Skåne, Lund, Sweden
^3^Oncology, Department of Clinical Sciences in Lund, Lund University, Lund, Sweden
^4^Hematology, Oncology and Radiation Physics, Region Skåne, Malmö, Sweden
^5^Clinical Genetics, Department of Laboratory Medicine, Lund University, Lund, Sweden

*Corresponding author: annelie.augustinsson@med.lu.se
